# Six-Gene Signature Associated with Immune Cells in the Progression of Atherosclerosis Discovered by Comprehensive Bioinformatics Analyses

**DOI:** 10.1155/2020/1230513

**Published:** 2020-07-25

**Authors:** Bin Zhao, Dan Wang, Yanling Liu, Xiaohong Zhang, Zheng Wan, Jinling Wang, Ting Su, Linshan Duan, Yan Wang, Yuehua Zhang, Yilin Zhao

**Affiliations:** ^1^Department of Oncology and Vascular Interventional Radiology, Zhongshan Hospital Affiliated to Xiamen University, Xiamen, Fujian, China; ^2^School of Medicine, Xiamen University, Xiamen, Fujian, China; ^3^School of Pharmaceutical Sciences, Xiamen University, Xiamen, Fujian, China; ^4^Department of Emergency, Zhongshan Hospital, Xiamen University, Xiamen, Fujian, China; ^5^Department of Ophthalmology, Howe Laboratory, Massachusetts Eye and Ear, Harvard Medical School, Boston 02114, USA; ^6^Medical Reproductive Auxiliary Specialty, People's Hospital of Jiuquan City, Gansu, China; ^7^Laboratory Animal Center, Xiamen University, Xiamen, Fujian, China

## Abstract

**Background:**

As a multifaceted disease, atherosclerosis is often characterized by the formation and accumulation of plaque anchored to the inner wall of the arteries and causes some cardiovascular diseases and vascular embolism. Numerous studies have reported on the pathogenesis of atherosclerosis. However, fewer studies focused on both genes and immune cells, and the correlation of genes and immune cells was evaluated via comprehensive bioinformatics analyses.

**Methods:**

29 samples of atherosclerosis-related gene expression profiling, including 16 human advanced atherosclerosis plaque (AA) and 13 human early atherosclerosis plaque (EA) samples from the Gene Expression Omnibus (GEO) database, were analyzed to get differentially expressed genes (DEGs) and the construction of protein and protein interaction (PPI) networks. Besides, we detected the relative fraction of 22 immune cell types in atherosclerosis by using the deconvolution algorithm of “cell type identification by estimating relative subsets of RNA transcripts (CIBERSORT).” Ultimately, based on the significantly changed types of immune cells, we executed the correlation analysis between DEGs and immune cells to discover the potential genes and pathways associated with immune cells.

**Results:**

We identified 17 module genes and 6 types of significantly changed immune cells. Correlation analysis showed that the relative percentage of T cell CD8 has negative correlation with the *C1QB* expression (*R* = −0.63, *p* = 0.02), and the relative percentage of macrophage M2 has positive correlation with the *CD86* expression (*R* = 0.57, *p* = 0.041) in EA. Meanwhile, four gene expressions (*CD53*, *C1QC*, *NCF2*, and *ITGAM*) have a high correlation with the percentages of T cell CD8 and macrophages (M0 and M2) in AA samples.

**Conclusions:**

In this study, we suggested that the progression of atherosclerosis might be related to *CD86*, *C1QB*, *CD53*, *C1QC*, *NCF2*, and *ITGAM* and that it plays a role in regulating immune-competent cells such as T cell CD8 and macrophages M0 and M2. These results will enable studies of the potential genes associated with immune cells in the progression of atherosclerosis, as well as provide insight for discovering new treatments and drugs.

## 1. Background

Atherosclerosis is a multifaceted, progressive, and chronic inflammatory arterial disease that is recognized to be the leading cause of morbidity and mortality around the world [1]). It is characterized by the formation and build-up of atherosclerosis plaque inside the damaged arteries [2, 3]. Plaque is composed of low-density lipoprotein (LDL) cholesterol, fat, calcium, and other substances existing in the blood, which can harden and narrow the arteries [4–7]. Many studies have shown that atherosclerosis can affect any arterial blood vessels in the body and can lead to the atherosclerosis-related diseases, including ischemic heart, carotid artery, peripheral artery, and chronic kidney diseases [8–13]. Risk factors include high blood pressure, abnormal cholesterol levels, diabetes, obesity, family genetic history, smoking, age, and an unhealthy lifestyle.

Data mining has been used in various applications, including sequencing [14], microarray gene expression analysis [15–17], single-nucleotide polymorphism detection [18, 19], and genomic loss and amplification (copy number variation) analysis [20, 21]. Using microarrays, integrated bioinformatics enables researchers to quickly identify differentially expressed target genes between atherosclerosis samples in a single experiment [22, 23]. CIBERSORT is a deconvolution computational method for quantifying immune cell fractions from bulk tissue gene expression profiles. This method can accurately calculate the relative proportion of 22 types of immune cell compositions in lesion samples [24, 25].

The detailed mechanism of the pathogenesis of atherosclerosis is unclear. Although studies have revealed that chronic inflammation can drive atherosclerosis, which is the leading cause of cardiovascular disease as confirmed by molecular and cellular experiments, fewer studies have been conducted to analyze the correlation of genes and immune cells in atherosclerosis-related big data.

In this study, we reanalyzed the GSE28829 dataset reported previously in Doring et al.'s team research [2] and detected potential target genes for atherosclerosis treatment from the perspective of big data analysis. We firstly identified candidate DEGs and significant immune cells. Then, we explored the correlation between gene expressions and the relative percentages of immune cells to identify potential gene signatures useful for the diagnosis and therapeutic treatment of atherosclerosis.

## 2. Materials and Methods

### 2.1. Data Acquisition

**Figure 1:**
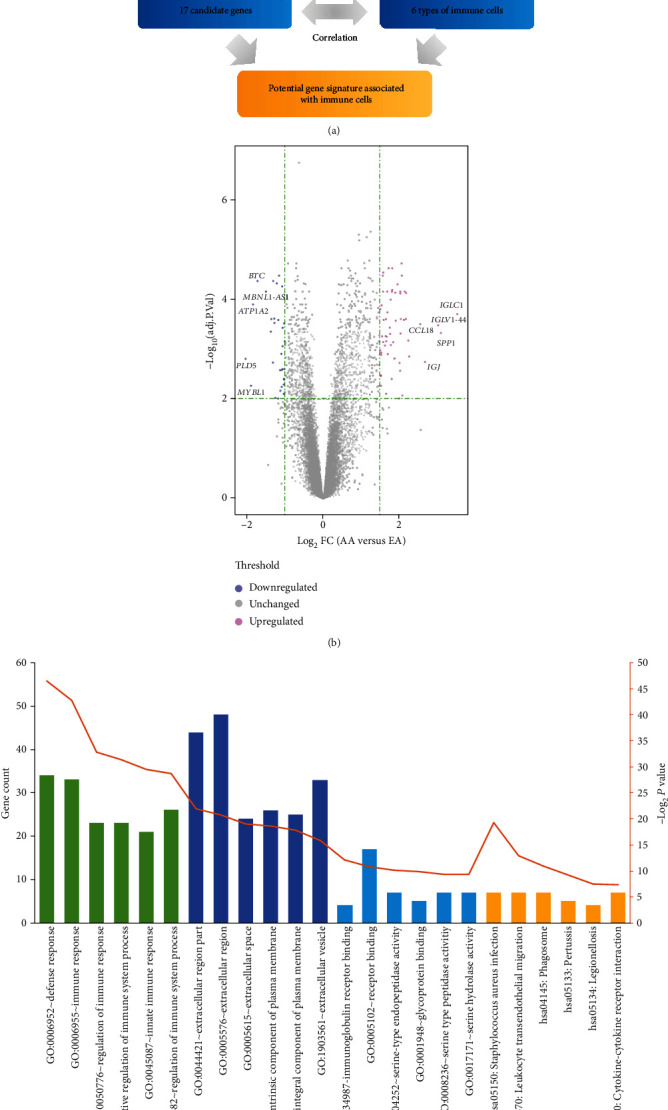
(a) Workflow of the analysis. (b) Volcano plot of differentially expressed genes; red represents upregulated genes, whereas blue represents downregulated genes. (c) Significance of GO and pathway enrichment of DEGs.

The robust multiarray averaging normalized microarray expression profile GSE28829 [2] and affiliated annotation file were downloaded from the National Center Biotechnology Information Gene Expression Omnibus (https://www.ncbi.nlm.nih.gov/geo/) website [26], which was tested on the GPL570 platform based on the Affymetrix Human Genome U133 Plus 2.0 array. GSE28829 contains 13 early atherosclerotic plaque samples (EA group) and 16 advanced atherosclerotic plaque samples (AA group) from the human carotid artery.  Figure 1(a) provides an overview of the analysis workflow.

### 2.2. Data Preprocessing

After the GSE28829 expression matrix was downloaded, probe identification was matched to the corresponding gene symbol. For multiprobes to one gene, we retained the probe showing a significant gene expression value after deleting the non-mRNA probe. Based on this gene expression matrix information, we identified the significant differentially expressed genes and immune cells.

### 2.3. Identification of DEGs

The *limma* package was utilized to identify differentially expressed genes (DEGs) between advanced atherosclerotic plaques and early atherosclerotic plaque samples in RStudio [27–29]. The criteria were as follows: (1) the adjusted *p* < 0.01, a moderate *t*-test corrected by Benjamini and Hochberg's method [30]; (2) log fold change (FC) of upregulated genes ≥ 1.5 or log fold change (FC) of downregulated genes ≤ −1.

### 2.4. Gene Ontology and Pathway Analysis

Gene Ontology (GO) is used to describe the roles of genes and gene products in any organism based on existing biological knowledge and is divided into three independent branches: biological process (BP), cellular component (CC), and molecular function (MF) (Harris et al., 2004; Harris et al., 2006). Metabolic pathways and gene signaling networks based on available databases such as KEGG [31] and Reactome [32] were used to describe the pathway enrichment analyses. We used the DAVID website (https://david. http://ncifcrf.gov/) for gene annotation and visualization to perform the GO and pathway analysis. *p* < 0.05, calculated via Fisher's exact test [33], was used as the threshold for statistical significance [32, 34].

### 2.5. PPI Network Construction and Module Analysis

First, the identified DEGs were uploaded to the STRING [35] (version 11.0) website which includes 2 billion interactions associated with 24.6 million proteins referred to 5090 organs. STRING was used to determine the PPIs between DEG-encoded proteins. Second, the minimum interaction score was set to 0.4. The PPI networks were constructed using Cytoscape software [36]. The built-in Molecular Complex Detection (MCODE), a well-known automated method for detecting highly interconnected subgraphs as molecular complexes or clusters in large PPI networks was utilized to screen the modules in the PPI network. The correlated parameter criteria were set by default, except *K*‐core = 7. Moreover, functional enrichment analysis was performed for DEGs in the significant module with *p* < 0.05, calculated via Fisher's exact test [34], as the cutoff criterion.

### 2.6. Immune Cell Infiltration Analysis

Normalized gene expression data were utilized to evaluate the relative proportions of 22 types of infiltrating immune cells via using the CIBERSORT algorithm [25]. The gene expression matrix was uploaded to the CIBERSORT online website (https://cibersort.stanford.edu) by setting the default signature matrix at 1000 permutations. CIBERSORT is a deconvolution algorithm that depends on a set of reference gene expression values (a “signature matrix” of 547 genes in 22 types of immune cells). Next, significant immune cells between EA and AA samples were identified with the threshold *Wilcoxon test* at *p* < 0.05.

### 2.7. Correlation Analysis of Genes and Immune Cells

Pearson correlation test analysis was carried out to illustrate the relationship between gene expressions and the relative percentages of immune cells in EA and AA samples, respectively [37]. The value of the correlation coefficient between gene expressions and the relative proportion of immune cells could indicate the strong, weak, or no correlation. Based on the paired *t*-test, *p* < 0.05 was considered statistically significant.

### 2.8. Statistical Analysis

The moderate *t*-test was used to identify differentially expressed genes. Fisher's exact test was applied to perform GO and KEGG analysis. The Wilcoxon test was applied to immune cell analysis. Paired *t*-test was applied to correlation analysis between genes and cells. All statistical analyses were carried out in R version 3.5.2 software.

## 3. Results

### 3.1. Identification of DEGs

In the study, we identified 91 differentially expressed genes (DEGs) in the AA group compared to the EA group (Figure 1(b) and Table 1). Among them, 59 DEGs were upregulated (adjusted *p* < 0.01 and log FC ≥ 1.5), and the remaining 32 DEGs were downregulated (adjusted *p* < 0.01 and log FC ≤ −1).

### 3.2. GO and Pathway Analysis

DEGs were uploaded to the DAVID website to identify the GO and pathway terms. As shown in Figure 1(c), significantly enriched GO and pathway DEGs were involved in defense response (BP), extracellular space (CC), immunoglobulin receptor binding (MF), and *Staphylococcus aureus* infection (KEGG pathway). As shown in Table 2, the significant GO terms of upregulated DEGs were mainly enriched in defense response (BP), immune response (BP), and regulation of immune response (BP), while significant GO terms of downregulated DEGs mainly were enriched in muscle contraction (BP), muscle system process (BP), and movement of cell or subcellular component (BP). The pathway terms of upregulated DEGs were enriched in *Staphylococcus aureus* infection (KEGG), phagosome (KEGG), and leukocyte transendothelial migration (KEGG), while the pathways of downregulated DEGs were unavailable (Table 3).

### 3.3. PPI Network Construction and Module Analysis

**Figure 2:**
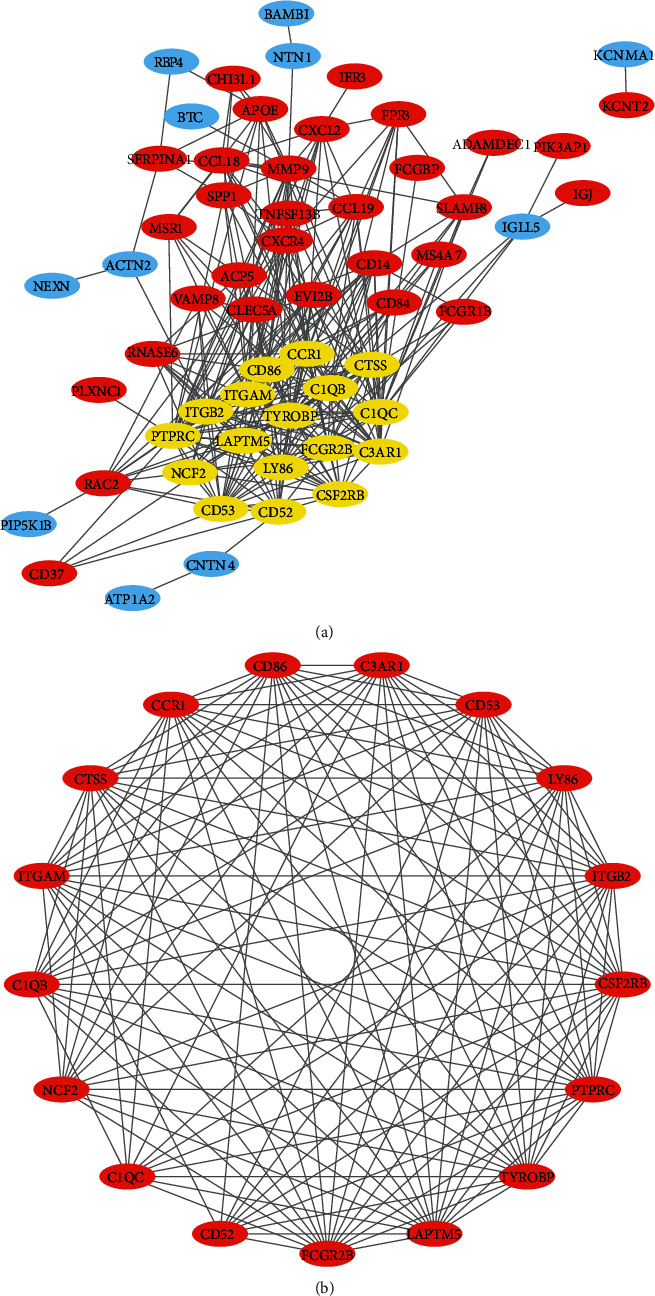
(a) Protein-protein interaction (PPI) networks; red represents upregulated genes, blue represents downregulated genes, and yellow represents the significant module genes. Analysis was performed with MCODE. (b) Significant module genes; red represents upregulated module genes.

After uploading the 91 DEGs into the STRING online database and downloading the TSV format file of the interaction of multiple genes to Cytoscape software for PPI network construction, 59 DEGs (48 upregulated and 11 downregulated genes) were filtered from the 91 DEGs to construct the PPI networks, which contained 59 nodes/genes and 306 edges (Figure 2(a)); 32 genes did not participate in the PPI networks. Among these 59 nodes/genes, 17 central nodes/genes in module 1 were identified by the MCODE app and significantly associated with immune system function (Figure 2(b), Figure S1).

### 3.4. Immune Cell Infiltration Analysis

**Figure 3:**
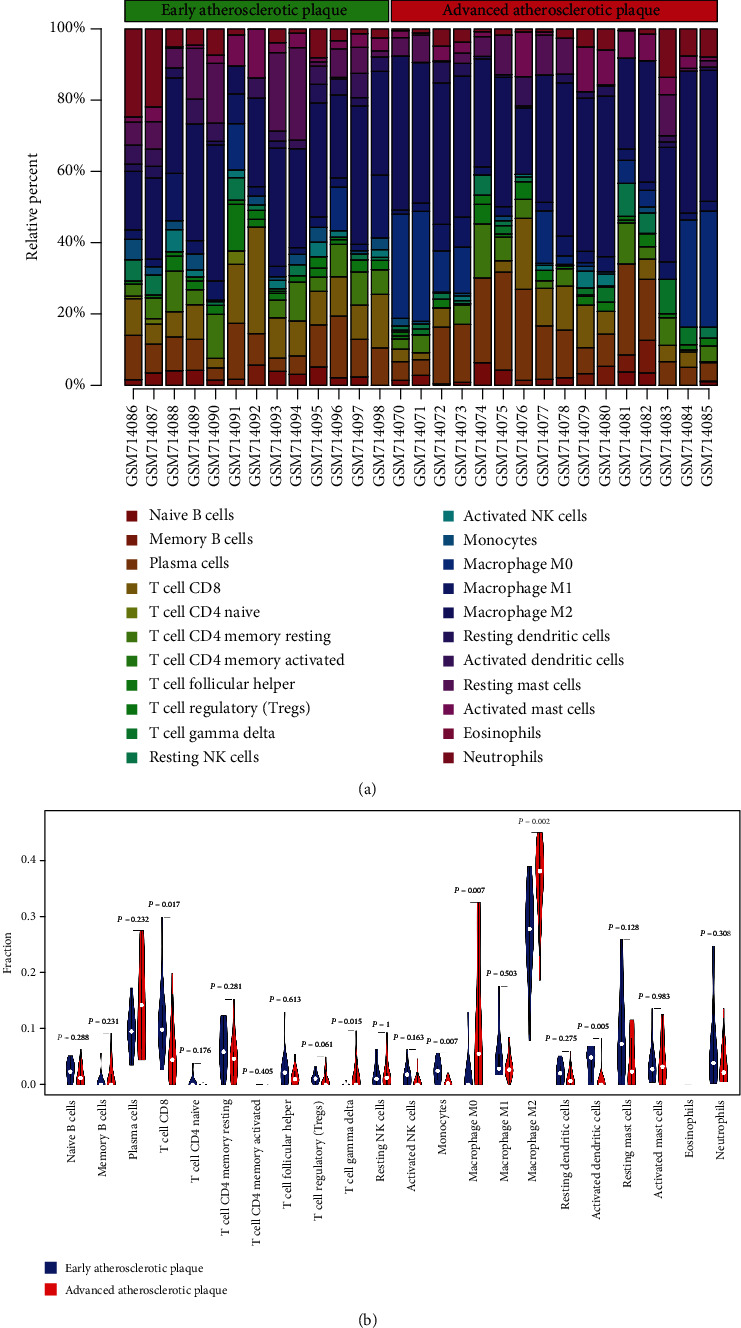
(a) Relative proportions of 22 types of infiltrated immune cells in EA and AA groups. (b) Significant changes in infiltrated immune cells in AA compared to EA group (Wilcoxon test *p* < 0.05).

We first used the CIBERSORT algorithm to investigate the relative proportion of the 22 subpopulations of immune cells among EA and AA samples (Figure 3(a)). The relative proportions of 6 types of immune cells were significantly different between the EA and AA groups (Figure 3(b)). The cell types were T cell CD8 (*p* = 0.017), T cell gamma delta (*p* = 0.015), monocytes (*p* = 0.007), macrophage M0 (*p* = 0.007), macrophage M2 (*p* = 0.002), and dendritic cells (activated) (*p* = 0.005). Among these 6 types of immune cells, we found that T cell CD8, monocytes, and dendritic cells (activated) in the EA group were present at higher fractions than in the AA group, while the other three types of immune cells showed the opposite results (Figure 3(b)).

### 3.5. Correlation Analysis of Genes and Immune Cells

**Figure 4:**
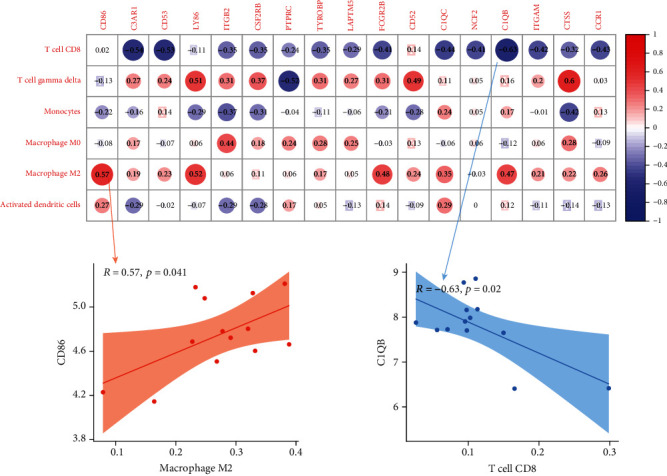
(a) Correlation between gene expressions and the relative percentages of immune cells in the EA group. (b) Scatterplots illustrate the exact relationship between the *CD86* expression and the relative proportion of macrophage M2 (*R* = 0.57, *p* = 0.041) and the correlation between the *C1QB* expression and the relative proportion of T cell CD8 (*R* = −0.63, *p* = 0.02). Gray-shaded areas in scatterplots represent the standard errors of the regression lines. *R*: correlation coefficient.

**Figure 5:**
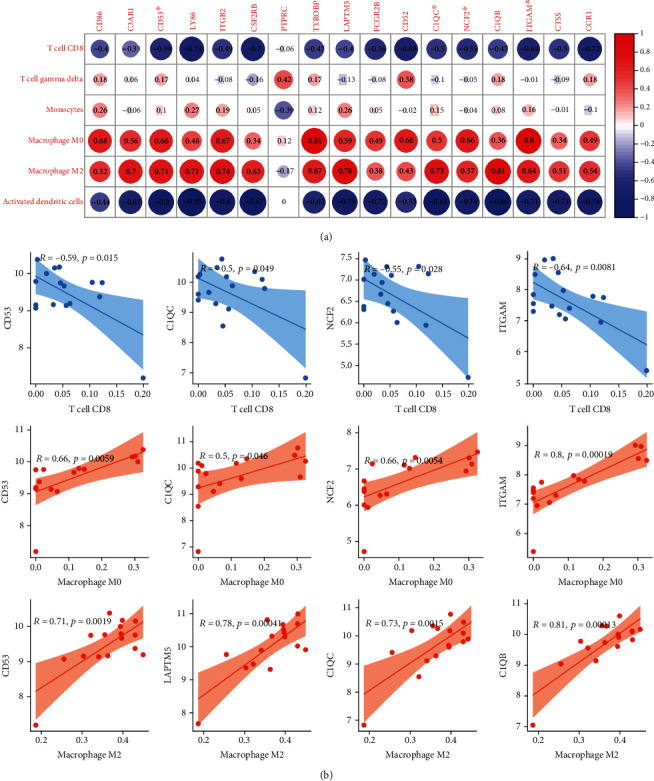
(a) Correlation between gene expressions and the relative percentages of immune cells in the AA group. (b) Scatterplots illustrate the relationship between four common gene expressions (*CD53*, *C1QC*, *NCF2*, and *ITGAM*) and the relative proportions of these three types of immune cells (T cell CD8, macrophage M0, and macrophage M2). Gray-shaded areas in scatterplots represent the standard errors of the regression lines. *R*: correlation coefficient. ^∗^Icon indicates the four common genes.

Correlation analysis (Pearson test) was carried out to illustrate and display the relationship between candidate genes and immune cells in EA and AA samples (Figures 4 and 5). As shown, *CD86* and macrophage M2 (*R* = 0.57, *p* = 0.041) and *C1QB* and T cell CD8 (*R* = −0.63, *p* = 0.02) represented good correlation (Figures 4(a) and 4(b)) in early atherosclerosis plaque samples. Moreover, most genes have a close correlation with immune cells in advanced atherosclerosis plaque samples. It is worth noting that four common genes (*CD53*, *C1QC*, *NCF2*, and *ITGAM*) from module 1 have a close correlation with T cell CD8 and macrophages M0 and M2 (Figures 5(a) and 5(b)). However, we have discarded that situation: although the *p* value was less than 0.05 and had a high absolute value of *R*, the scatter plot shows that the point distribution was aggregated to zero (supplement Figures S2-3).

## 4. Discussion

Atherosclerosis is a disease caused by plaque accumulation within the arteries. Current studies have confirmed that immune cells including dendritic cells, several T cells, monocyte/macrophage subsets, and neutrophils are associated with atherosclerosis [2, 38–40]. It has been shown that specific therapies targeting the pro/anti-inflammatory cytokines such as *CCL2*, *TNFα*, and *IL-6* have suggested slowing in the progression of atherosclerosis in animal models and might improve cardiovascular outcomes in human subjects in large-scale phase III trials. [41, 42]. Notably, the monoclonal antibody canakinumab, targeting to *IL-1β*, has reduced the risk of adverse cardiovascular events [41, 42].

In the current study, we aimed to identify the potential molecular gene signatures associated with the immune system during the progression of atherosclerotic disease. We first figured out the genes in module 1 (17 genes) and significantly changed types of immune cells (6 types of immune cells) between advanced atherosclerosis and early atherosclerosis. Afterward, according to the correlation analysis between genes and immune cells, we inferred that *CD86* and *C1QB* have a good correlation with macrophage M2 and T cell CD8 in EA, respectively. What is more, most of the genes have associated with T cell CD8, macrophages (M0 and M2), and four common genes (*CD53*, *C1QC*, *NCF2*, and *ITGAM*), and all have correlation with the three types of immune cells in AA.

*CD86* (cluster of differentiation 86) is a protein encoded by *CD86*, which is expressed on antigen-presenting cells (APCs) and provides costimulatory signals to T cells [43]. Meletta et al. used *CD86*/*CD80* as a probe for atherosclerosis imaging [43]. Transfer of native *Foxp3+* T cells showed a protective effect against experimental atherosclerosis (Ait-Oufella et al.; [44, 45]). *CD53* (leukocyte surface antigen) is a member of the “tetraspan family” of membrane proteins and is expressed on various immune cells [46, 47]. *CD53* can contribute to improving the transduction of *CD2*-generated signals in T cells and natural killer cells [48]. *C1QB* (complement *C1q* B chain) or *C1QC* (complement *C1q* C chain) encodes the C-chain or B-chain polypeptide of serum complement subcomponent *C1q*, respectively, and deficiency of *C1q* is associated with glomerulonephritis and lupus erythematosus. The Bos et al. team has suggested that *C1QB* might be associated with atherosclerosis and coronary artery disease [49]. What is more, the Khoonsari et al. group has revealed that the lower levels of *C1QB* and *C1QC* were involved in cell adhesion, migration, regulation of the synapse, and the immune system [50]. *NCF2* (neutrophil cytosol factor 2) encodes a subunit of *NADPH* oxidase, and mutation in this gene can result in chronic granulomatous disease [51]. However, no research has revealed that *NCF2* was involved in atherosclerosis. *ITGAM* (integrin alpha M) is known as complement receptor 3A (*CR3A*) or cluster of differentiation molecule 11B (*CD11B*) [52] and primarily expressed on the surface of innate immune cells [53]. Recent reports revealed that *NCF2* and *ITGAM* play significant immune-regulatory roles in autoimmune disease [54, 55]. To date, fewer studies were focused on how genes (*C1QB*, *C1QC*, *NCF2*, and *ITGAM*) regulate immune cells (T cell CD8 and macrophages M0 or M2) and their relationship between gene expressions and the relative percentages of immune cells.

Based on this fact, (1) atherosclerotic diseases are related to immune cells and (2) there are examples of researchers using modified genes (such as *IL-1β*, *CCL2*, and *IL-6*) to treat atherosclerotic diseases [41, 42]. The innovation of this study is to screen out gene signatures associated with immune cells in the progression of atherosclerosis. According to the relationship between gene expressions and the relative proportions of immune cells, these genes may interact with immune cells through some unknown cell membrane receptors or ligands. It may be possible to modify the interactions of these genes with membrane receptors or ligands to identify new therapeutic treatments for atherosclerosis, as well as mechanisms of atherosclerosis regulation.

Nevertheless, there exist some inevitable difficulties in this study that should be taken into consideration. For instance, the atherosclerosis-related datasets were fewer and not as easily acquired and collected from the open public database as cancer datasets, which leads to the lack of comprehensiveness of the study which cannot be used to verify our results. Although the limited sample size of atherosclerosis may reduce the confidence, the approaches and ideas in this study are helpful in enlightening the inspiration of other researchers. Of course, additional molecular and cellular experiments should be performed to assess their characteristics.

## 5. Conclusions

The identification of 6 specific gene signatures and correlated immune cells in the progression of atherosclerosis may give us a clue to explore the mechanism of cardiovascular disease and its therapeutic treatments.

## Figures and Tables

**Table 1:** The 91 differentially expressed genes were identified in AA samples compared to EA samples. (The differentially expressed genes were ranked from the smallest to the largest of adjusted *p* value).

DEGs	Gene name
Upregulated genes (logFC ≥ 1.5)	SLAMF8, SERPINA1, VAMP8, C3AR1, CD52, CD84, CCR1, FCGBP, CD14, FCGR1B, ITGB2, LAPTM5, PIK3AP1, C1QB, APOE, KYNU, CTSS, RAC2, CD37, TYROBP, IGLC1, ACP5, TNFSF13B, CD53, CCL19, LY86, NPL, CCL18, IGLV1-44, BCAT1, SPP1, FCGR2B, C1QC, FABP5, PTPRC, MS4A7, CHI3L1, PLXNC1, GIMAP2, IER3, ADAMDEC1, CSF2RB, ITGAM, NCF2, CEMIP, CLEC5A, IGKC, CD86, IGLL3P, IGJ, CXCR4, CXCL2, RNASE6, FPR3, MSR1, KCNT2, EVI2B, IGHM, MMP9
Downregulated genes (logFC ≤ −1)	ANGPTL1, TMEM35, BTC, BAG2, SLMAP, MBNL1-AS1, ATP1A2, PIP5K1B, C3orf70, SH3BGR, CNTN4, SBSPON, CAB39L, ACADL, ACTN2, NEXN, PDZRN3, PLD5, SLC22A3, KCNMA1, TTLL7, BAMBI, PPP1R1A, NTN1, AMIGO2, APCDD1, MYBL1, CNN1, RBP4, TOX2, CNTN1, LGR6

**Table 2:** The significant Gene Ontology enrichments of differentially expressed genes (DEGs).

Category	Term	Count	*p* value
*Upregulated genes*			
GOTERM_BP_FAT	GO:0006952~defense response	34	1.78*E*-22
GOTERM_BP_FAT	GO:0006955~immune response	32	9.60*E*-20
GOTERM_BP_FAT	GO:0050776~regulation of immune response	23	4.74*E*-15
GOTERM_BP_FAT	GO:0045087~innate immune response	21	1.47*E*-13
GOTERM_BP_FAT	GO:0002684~positive regulation of immune system process	22	1.68*E*-13
GOTERM_BP_FAT	GO:0002682~regulation of immune system process	25	2.27*E*-13
GOTERM_BP_FAT	GO:0050778~positive regulation of immune response	18	8.88*E*-12
GOTERM_BP_FAT	GO:0048584~positive regulation of response to stimulus	26	1.26*E*-10
GOTERM_BP_FAT	GO:0009605~response to external stimulus	26	1.99*E*-10
GOTERM_BP_FAT	GO:0002250~adaptive immune response	14	2.78*E*-10
GOTERM_BP_FAT	GO:0006954~inflammatory response	16	4.15*E*-10
GOTERM_BP_FAT	GO:0002253~activation of immune response	15	6.56*E*-10
GOTERM_BP_FAT	GO:0002764~immune response-regulating signaling pathway	14	4.79*E*-09
GOTERM_BP_FAT	GO:0007166~cell surface receptor signaling pathway	27	7.57*E*-09
GOTERM_BP_FAT	GO:0050900~leukocyte migration	12	1.43*E*-08
GOTERM_BP_FAT	GO:0002757~immune response-activating signal transduction	13	2.47*E*-08
GOTERM_BP_FAT	GO:0032101~regulation of response to external stimulus	14	1.75*E*-07
GOTERM_BP_FAT	GO:0002252~immune effector process	14	2.44*E*-07
GOTERM_CC_FAT	GO:0044421~extracellular region part	33	4.63*E*-07
GOTERM_CC_FAT	GO:0005615~extracellular space	20	4.84*E*-07
*Downregulated genes*			
GOTERM_BP_FAT	GO:0006936~muscle contraction	5	0.00187531
GOTERM_BP_FAT	GO:0003012~muscle system process	5	0.003885779
GOTERM_BP_FAT	GO:0006928~movement of cell or subcellular component	9	0.006091447
GOTERM_BP_FAT	GO:0015672~monovalent inorganic cation transport	5	0.007385488
GOTERM_BP_FAT	GO:0002028~regulation of sodium ion transport	3	0.007948344
GOTERM_BP_FAT	GO:0040011~locomotion	8	0.0093652
GOTERM_BP_FAT	GO:0043269~regulation of ion transport	5	0.011584817
GOTERM_BP_FAT	GO:0010959~regulation of metal ion transport	4	0.014867703
GOTERM_MF_FAT	GO:0003779~actin binding	4	0.017959842
GOTERM_BP_FAT	GO:0006812~cation transport	6	0.018744883
GOTERM_BP_FAT	GO:0030007~cellular potassium ion homeostasis	2	0.019293154
GOTERM_BP_FAT	GO:0042391~regulation of membrane potential	4	0.019968055
GOTERM_BP_FAT	GO:0034765~regulation of ion transmembrane transport	4	0.024929627
GOTERM_BP_FAT	GO:0034762~regulation of transmembrane transport	4	0.02729303
GOTERM_BP_FAT	GO:0055075~potassium ion homeostasis	2	0.028804816
GOTERM_MF_FAT	GO:0008092~cytoskeletal protein binding	5	0.029503547
GOTERM_BP_FAT	GO:0071805~potassium ion transmembrane transport	3	0.034876856
GOTERM_BP_FAT	GO:0071804~cellular potassium ion transport	3	0.034876856
GOTERM_BP_FAT	GO:0032412~regulation of ion transmembrane transporter activity	3	0.03593287
GOTERM_BP_FAT	GO:0048738~cardiac muscle tissue development	3	0.036287643

**Table 3:** The significant signal pathways of differentially expressed genes (DEGs).

Pathway	Term	Count	*P* value
*Upregulated genes*			
KEGG_PATHWAY	hsa05150: Staphylococcus aureus infection	7	2.40*E*-07
KEGG_PATHWAY	hsa04145: phagosome	7	9.36*E*-05
KEGG_PATHWAY	hsa04670: leukocyte transendothelial migration	6	2.60*E*-04
KEGG_PATHWAY	hsa05133: pertussis	5	5.28*E*-04
KEGG_PATHWAY	hsa04060: cytokine-cytokine receptor interaction	7	0.001252687
KEGG_PATHWAY	hsa04062: chemokine signaling pathway	6	0.002299717
KEGG_PATHWAY	hsa05134: legionellosis	4	0.002506044
BioCarta	h_blymphocytePathway: B lymphocyte cell surface molecules	3	0.003628217
KEGG_PATHWAY	hsa04610: complement and coagulation cascades	4	0.005025012
KEGG_PATHWAY	hsa05323: rheumatoid arthritis	4	0.009854418
KEGG_PATHWAY	hsa05152: tuberculosis	5	0.011906527
KEGG_PATHWAY	hsa04672: intestinal immune network for IgA production	3	0.023560079
KEGG_PATHWAY	hsa04380: osteoclast differentiation	4	0.028350582
KEGG_PATHWAY	hsa05416: viral myocarditis	3	0.033703679
KEGG_PATHWAY	hsa04514: cell adhesion molecules (CAMs)	4	0.034833291
*Downregulated genes*			

No significant signal pathway (*P* value < 0.05) available.

## Data Availability

The data associated with this article has been deposited in the NCBI-GEO website (https://www.ncbi.nlm.nih.gov/geo/query/acc.cgi).

## Abbreviations

NCBI: National Center for Biotechnology Information
GEO: Gene Expression Omnibus
DEGs: Differentially expressed genes
PPI: Protein and protein interaction
CIBERSORT: Cell type identification by estimating relative subsets of RNA transcripts
LDL: Low-density lipoprotein
EA: Early atherosclerosis
AA: Advanced atherosclerosis
GO: Gene Ontology
KEGG: Kyoto Encyclopedia of Genes and Genomes
BP: Biological process
CC: Cellular component
MF: Molecular function
MCODE: Molecular complex detection.

## Supplementary Materials

Supplementary Materials Figure S1: the chord plot for functional enrichments of module 1 genes. Figure S2: the scatterplot of correlation between CTSS expression and the relative proportion of T cell gamma delta. Gray-shaded areas in scatterplots represent the standard errors of the blue regression lines. *R*: correlation coefficient. Figure S3: the scatterplot of correlation between 17 gene expressions and the proportion of dendritic cells activated. Gray-shaded areas in scatterplots represent the standard errors of the blue regression lines. *R*: correlation coefficient.
