# Supplementary figures and images for: Six-Gene Signature Associated with Immune Cells in the Progression of Atherosclerosis Discovered by Comprehensive Bioinformatics Analyses
Source: Cardiovasc Ther. 2020 Jul 25;2020:1230513. doi: 10.1155/2020/1230513 (PMC7416237; doi:10.1155/2020/1230513)

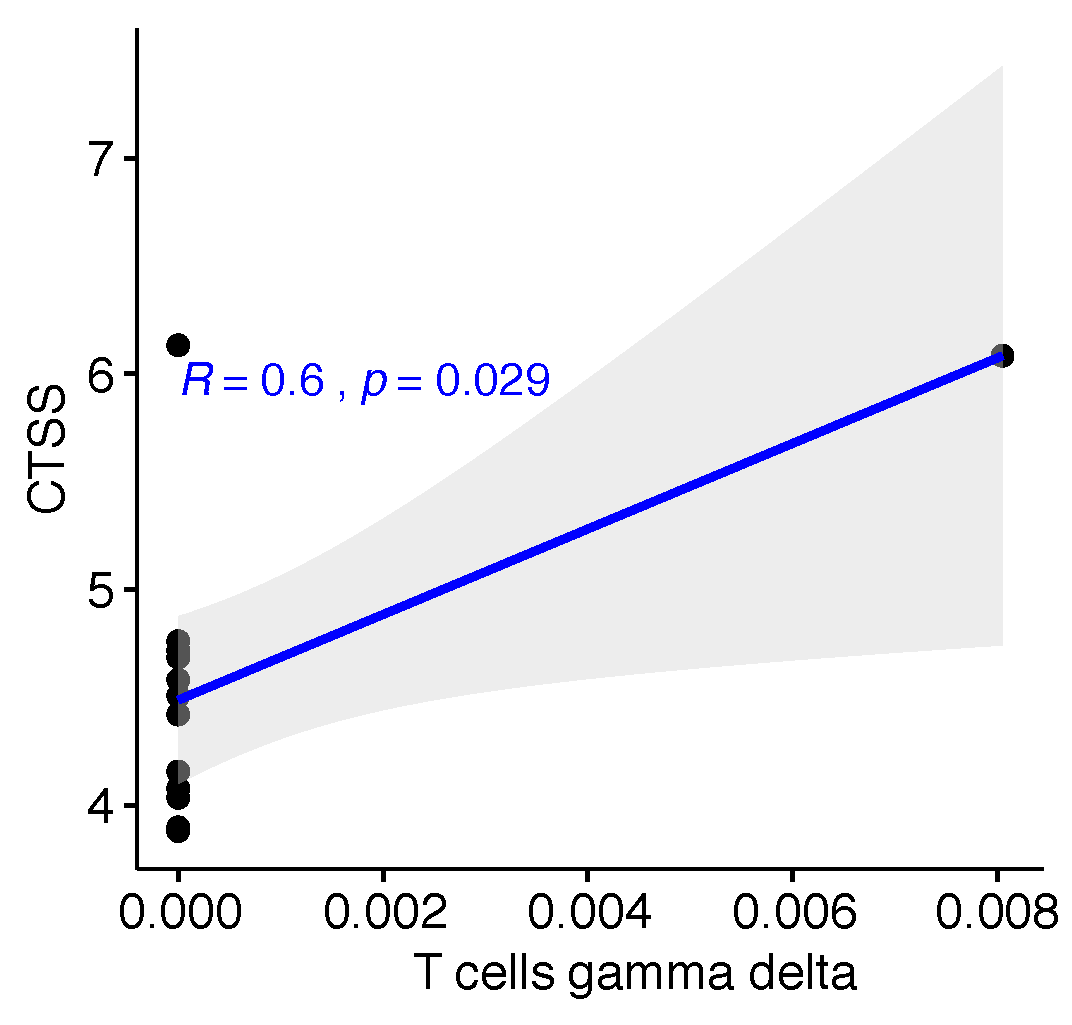

Supplement: Supplementary Materials — Figure S1: the chord plot for functional enrichments of module 1 genes. Figure S2: the scatterplot of correlation between CTSS expression and the relative proportion of T cell gamma delta. Gray-shaded areas in scatterplots represent the standard errors of the blue regression lines. R: correlation coefficient. Figure S3: the scatterplot of correlation between 17 gene expressions and the proportion of dendritic cells activated. Gray-shaded areas in scatterplots represent the standard errors of the blue regression lines. R: correlation coefficient. [file 1230513.f1.zip › Figure S2.tif]

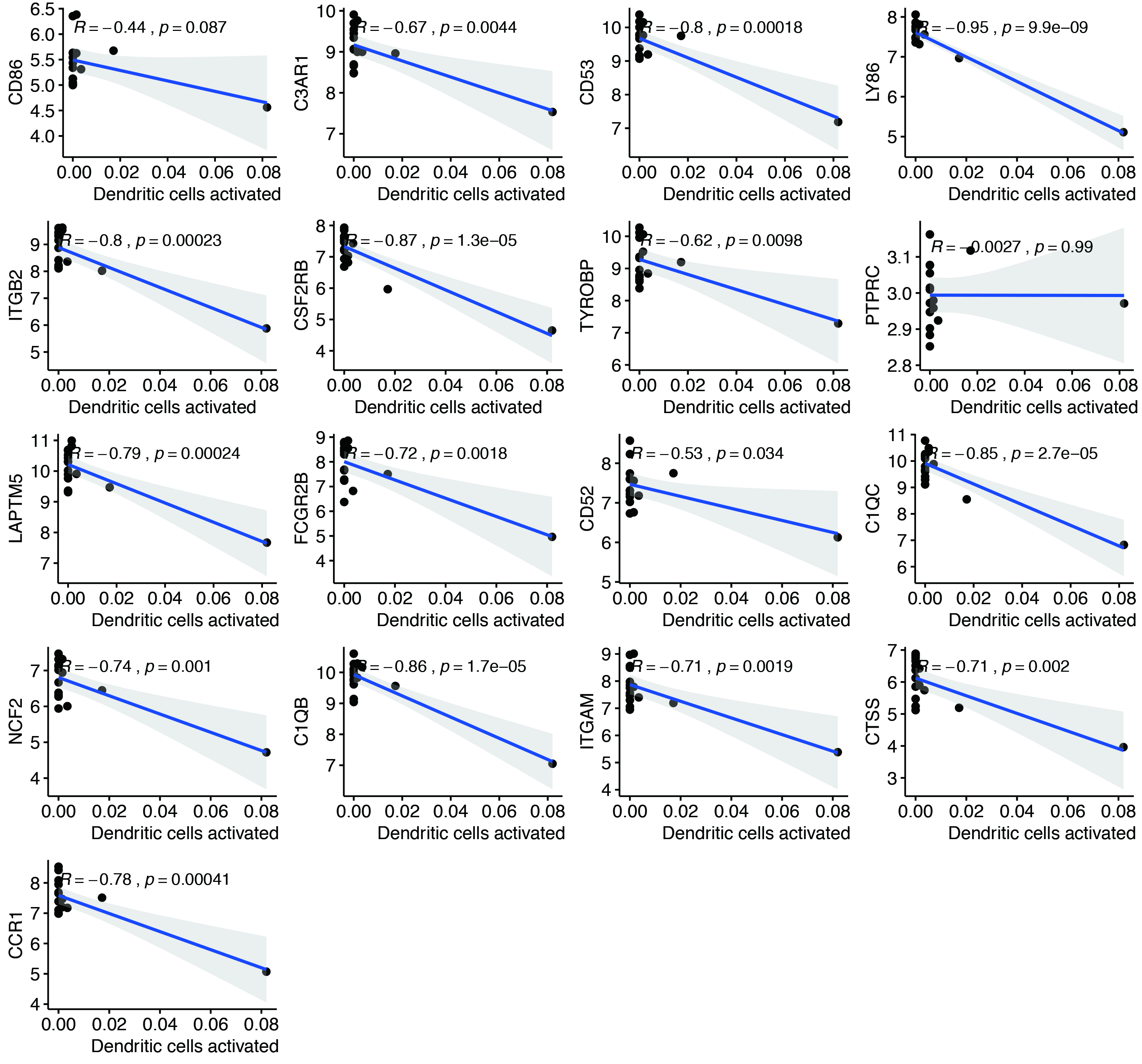

Supplement: Supplementary Materials — Figure S1: the chord plot for functional enrichments of module 1 genes. Figure S2: the scatterplot of correlation between CTSS expression and the relative proportion of T cell gamma delta. Gray-shaded areas in scatterplots represent the standard errors of the blue regression lines. R: correlation coefficient. Figure S3: the scatterplot of correlation between 17 gene expressions and the proportion of dendritic cells activated. Gray-shaded areas in scatterplots represent the standard errors of the blue regression lines. R: correlation coefficient. [file 1230513.f1.zip › Figure S3.tif]
